# Persistent stromal fibroblast activation is present in chronic tendinopathy

**DOI:** 10.1186/s13075-016-1218-4

**Published:** 2017-01-25

**Authors:** Stephanie G. Dakin, Christopher D. Buckley, Mohammad Hussein Al-Mossawi, Robert Hedley, Fernando O. Martinez, Kim Wheway, Bridget Watkins, Andrew J. Carr

**Affiliations:** 1 Nuffield Department of Orthopaedics, Rheumatology and Musculoskeletal Sciences, Botnar Research Centre, University of Oxford, Nuffield Orthopaedic Centre, Headington, OX3 7LD UK; 2 Rheumatology Research Group Institute of Inflammation and Ageing, University of Birmingham research laboratories, Queen Elizabeth Hospital, Birmingham, UK; 3 Faculty of Health & Medical Sciences, University of Surrey, Guildford, GU2 7XH UK

**Keywords:** Tendon, Tendinopathy, Inflammation, Stromal fibroblast

## Abstract

**Background:**

Growing evidence supports a key role for inflammation in the onset and progression of tendinopathy. However, the effect of the inflammatory infiltrate on tendon cells is poorly understood.

**Methods:**

We investigated stromal fibroblast activation signatures in tissues and cells from patients with tendinopathy. Diseased tendons were collected from well-phenotyped patient cohorts with supraspinatus tendinopathy before and after sub-acromial decompression treatment. Healthy tendons were collected from patients undergoing shoulder stabilisation or anterior cruciate ligament repair. Stromal fibroblast activation markers including podoplanin (PDPN), CD106 (VCAM-1) and CD248 were investigated by immunostaining, flow cytometry and RT-qPCR.

**Results:**

PDPN, CD248 and CD106 were increased in diseased compared to healthy tendon tissues. This stromal fibroblast activation signature persisted in tendon biopsies in patients at 2–4 years post treatment. PDPN, CD248 and CD106 were increased in diseased compared to healthy tendon cells. IL-1β treatment induced PDPN and CD106 but not CD248. IL-1β treatment induced NF-κB target genes in healthy cells, which gradually declined following replacement with cytokine-free medium, whilst *PDPN* and *CD106* remained above pre-stimulated levels. IL-1β-treated diseased cells had more profound induction of *PDPN* and *CD106* and sustained expression of *IL6* and *IL8* mRNA compared to IL-1β-treated healthy cells.

**Conclusions:**

We conclude that stromal fibroblast activation markers are increased and persist in diseased compared to healthy tendon tissues and cells. Diseased tendon cells have distinct stromal fibroblast populations. IL-1β treatment induced persistent stromal fibroblast activation which was more profound in diseased cells. Persistent stromal fibroblast activation may be implicated in the development of chronic inflammation and recurrent tendinopathy. Targeting this stromal fibroblast activation signature is a potential therapeutic strategy.

## Background

Musculoskeletal diseases account for 5 of the top 15 causes of years lived with disease in well-resourced health systems [1]. Soft-tissue pathologic conditions such as tendinopathy are a common cause of pain and loss of function and an important and increasing component of health expenditure in ageing societies [2]. Diseased tendons heal by forming a repair scar; however, the normal architecture, composition, and tissue function are not fully restored, increasing susceptibility to chronic injury. The aetiology of tendinopathy is complex and multifactorial, encompassing the effects of repetitive damage, daily exercise, ageing and genetic factors [3, 4]. Growing evidence supports the contribution of inflammation to the onset and progression of tendinopathy [5–8]; however, the mechanisms underpinning the development of chronic tendon inflammation are poorly understood.

Recent work highlights the complex activation states of immune cells including macrophages populating diseased human shoulder tendons [8]. Investigation of inflammation activation pathways in cultured stromal cells from diseased human tendons has revealed that diseased stromal cells may be primed for inflammation [8]. However, the mechanism of this priming and the relative contribution of tendon stromal cells to sustaining chronic inflammation are unknown.

Recent studies support the importance of tissue microenvironments and the innate immune response in perpetuating the inflammatory process. Non-myeloid/non-lymphoid populations such as resident stromal fibroblasts are known to play a prominent role in the generation and maintenance of chronic synovial inflammation [9, 10]. Stromal fibroblast activation is reported in rheumatoid arthritis (RA) in which resident stromal cells fail to switch off their inflammatory programme. Phenotypic alterations in RA synovial fibroblasts play an important role in the switch from resolving to persistent disease [11, 12].

The process by which fibroblasts produce cytokines, chemokines, prostanoids and extracellular matrix proteins is termed “fibroblast activation”. Activated fibroblasts are found in damaged, inflamed or healing tissues and promote the retention of immune cells and regulate their behaviour [12]. Previously identified stromal fibroblast activation markers in the synovium in RA include podoplanin (PDPN), CD106 (VCAM-1) and CD248 (tumour endothelial marker-1/endosialin). PDPN is a transmembrane glycoprotein implicated in the invasiveness of cancer metastasis and CD106 functions as a cell adhesion molecule [13]. CD248 is a transmembrane receptor with ligands that include collagen 1 and fibronectin [14]. CD248 expression is up-regulated by inflammation, fibrosis, angiogenesis and malignancy [15, 16]. These stromal fibroblast activation markers have been identified in different locations of the synovium in RA. PDPN and CD106 are located in the synovial lining and CD248 in the sub-lining layer, and they are thought to represent distinct fibroblast subsets [12, 13].

In this study, we identified the potential role of tendon stromal fibroblasts (resident fibroblasts populating tendons) as an important tissue-resident population implicated in the development of chronic tendon inflammation. We studied supraspinatus tendon tissues from a well-phenotyped longitudinal cohort of patients with symptoms pre and post treatment. We characterised distinct stromal fibroblast activation signatures in healthy, diseased and post-treatment tendon tissues. As IL-1β induces NF-κB target genes known to be highly expressed in early-stage tendinopathy [8], we further investigated if IL-1β treatment of cultured tendon cells induces persistent stromal fibroblast activation, and if this response differs between healthy and diseased cells. We hypothesized that diseased tendons express stromal fibroblast activation markers and that IL-1β-treated diseased tendon cells show profound induction of PDPN, CD106 and NF-κB target genes.

## Methods

### Collection of tendon tissues

Patients were recruited from shoulder referral clinics where the structural integrity of the rotator cuff was determined by ultrasound. Patients presenting to the referral shoulder clinic had not responded to non-surgical treatment, including a course of physical therapy and glucocorticoid injections into the sub-acromial space, and had experienced pain for a minimum of 3 months. Patients completed the Oxford shoulder score (OSS), a validated and widely used clinical outcome measure scoring from 0 (severe disease) to 48 (normal function) [17]. Samples of healthy supraspinatus tendons (n = 5) were collected intra-operatively from patients undergoing shoulder surgery for post-traumatic instability. These biopsies were collected from male and female patients (ages 20–30 years, mean 23 ± 3.8 years) who had intact supraspinatus tendons on ultrasound, which was confirmed at surgery. Healthy subscapularis tendons (n = 4) were collected from male or female patients undergoing shoulder surgery for post-traumatic instability (ages 61–77 years, mean 66 ± 8 years). Body mass index (BMI) in the patients with healthy shoulder tendons was 24.5 (±1.5).

Diseased supraspinatus tendons were collected from male and female patients undergoing sub-acromial decompression surgery (biopsies were collected from six patients) or surgical debridement of a supraspinatus tendon tear (n = 9). Tendon tear sizes were classified as small (≤1 cm), medium (>1 and ≤3 cm), large (>3 and ≤5 cm) and massive (>5 cm in anterior-posterior length) [18]. Patients with diseased supraspinatus tendons were aged between 44 and 75 years (mean 55 ± 18.3 years). BMI in the patients with diseased tendons was not significantly different to that in the healthy group (27.8 ± 1.2).

Torn tendons were collected under research ethics from the Oxford Musculoskeletal Biobank (09/H0606/11). Biopsies were also taken from patients between 2 and 4 years after undergoing sub-acromial decompression surgery, in whom pain had resolved completely (n = 6) or pain persisted (n = 5). Post-treatment biopsies were collected by percutaneous ultrasound-guided biopsy under local anaesthesia. The biopsy specimen was taken using a Trucut needle 5–10 mm posterior to the anterior edge of the supraspinatus tendon. This validated biopsy technique is described in detail elsewhere [19]. Exclusion criteria for all patients in this study included previous shoulder surgery, other pathologic conditions of the shoulder or acute trauma, rheumatoid arthritis and systemic inflammatory disease.

For cell experiments, healthy hamstring tendons were collected from 10 male and female patients undergoing surgical reconstruction of their anterior cruciate ligament. All patients were aged between 18 and 48 years (mean 25.5 ± 11 years). BMI in the patients with healthy hamstring tendons was 24.9 (±2.1) and was not significantly different to that in the diseased patient group. Hamstring tendons were collected under research ethics from the Oxford Musculoskeletal Biobank (09/H0606/11). Hamstring tendons were immediately placed in Dulbecco’s minimum essential medium (DMEM)/F12 (Lonza) and processed in tissue culture to isolate the tendon-derived stromal cells.

### Processing of tendon samples

#### Immunohistochemistry analysis and immunofluorescence

Healthy and diseased supraspinatus tendons were immersed in 10% buffered formalin for 0.5 mm/hour. After fixation, tendons were processed using a Leica ASP300S tissue processor and embedded in paraffin wax. Tissues were sectioned at 4 μm using a rotary RM2135 microtome (Leica Microsystems Ltd.) onto adhesive glass slides and baked at 60 °C for 30 minutes and 37 °C for 60 minutes.

#### Histological assessment of healthy and diseased supraspinatus tendons

Histological assessment of tendons collected from the study cohort was performed on hematoxylin-and-eosin-stained sections using the Bonar scoring system (0–12) that evaluates tissue structure [20]. Healthy supraspinatus tendons exhibited a more normal tissue architecture (median 2, interquartile range 1–2) compared to tendinopathic (median 7, interquartile range 6–8) and torn supraspinatus tendons (median 10, interquartile range 8.25–10).

#### Gene expression

Samples of healthy subscapularis and diseased supraspinatus tendons were immediately snap-frozen in liquid nitrogen and stored at −80 °C until RNA extraction.

### Immunohistochemistry and immunofluorescence for identification of stromal fibroblast activation markers in tendons

For antigen retrieval, slides were baked at 60 °C for 60 minutes, and tissue sections subjected to deparaffinization and target retrieval steps (heat-mediated antigen retrieval at high pH) using an automated PT Link (Dako). For single-staining immunohistochemistry analysis, antibody staining was performed using the EnVision FLEX visualization system with an Autostainer Link 48 (Dako). Antibody binding was visualized using FLEX 3,3’-diaminobenzidine (DAB) substrate working solution and hematoxylin counterstain (Dako) using the recommended manufacturer protocols. After staining, slides were taken through graded alcohol and xylene and mounted in Pertex mounting medium (Histolab). For multiple antibody immunofluorescence staining and image acquisition, protocols were adapted from Dakin et al., 2015 [8], using the primary antibodies listed in Table 1. Sections of diseased rheumatoid synovium were used as positive controls to confirm immunostaining for PDPN and CD248. Isotype control antibodies were a cocktail of mouse immunoglobulin G (IgG_1_), IgG_2a_, IgG_2b_, IgG_3_, and IgM (Dako) and rabbit immunoglobulin fraction of serum from non-immunized rabbits, solid-phase absorbed (Dako).

**Table 1:** Primary antibodies used for immunohistochemistry analysis and immunofluorescence

Antibody	Clone	Isotype	Species	Dilution
Podoplanin (PDPN) Abcam Ab10288	18H5	IgG1	Mouse	1:100
CD248 (TEM1) Abcam Ab204914	EPR17081	IgG	Rabbit monoclonal	1:1000
CD106 (VCAM-1) LS-Biosciences LS_C313019		IgG	Rabbit polyclonal	1:100
TLR4 Abcam Ab22048	76B357.1	IgG2b	Mouse	1:200

### Isolation of tendon-derived stromal cells

Tendon cells were isolated from healthy hamstring and diseased supraspinatus tendons. Diseased tendon cells were isolated from patients with small to medium tendon tears (<3 cm in length). Small to medium tendon tears are known to express genes and proteins induced by interferon and NFκB inflammation activation pathways [8]. Tendons were cut into 2-mm^3^ explants and incubated in DMEM/F12 (Lonza) containing 50% fetal calf serum (FCS; Labtech) and 1% penicillin-streptomycin (Lonza). Fresh medium was replaced every 4 days, and cells were allowed to grow out from explants over time in a tissue culture incubator at 37 °C and 5% CO_2_. Once cells were confluent, explants were removed and media replaced with DMEM/F12 containing 10% FCS and 1% penicillin-streptomycin. Cells between passages 1 and 3 were used for all experiments.

### Treatment of tendon-derived stromal cells with IL-1β

Tendon-derived stromal cells isolated from healthy hamstring and diseased supraspinatus were seeded at a density of 30,000 cells per well in a 12-well plate (mRNA) or 60,000 cells in a 6-well plate (flow cytometry). Cells were allowed to reach 80% confluence prior to stimulation with IL-1β (10 ng/mL^-1^). Tendon cells were incubated in DMEM F12 medium (Lonza) containing 1% heat-inactivated human serum (Sigma). Medium containing sterile filtered 0.1% endotoxin-free BSA (Sigma) diluted in PBS was used for vehicle-only controls. After IL-1β or vehicle treatment, cells were incubated for 24 hours at 37 °C and 5% CO_2_ until harvest of the lysate for mRNA or flow cytometry.

### Extraction of RNA from tendons

Protocols for RNA extraction from healthy and diseased tendon tissues and cells, complementary DNA synthesis and quantitative polymerase chain reaction are described elsewhere [8]. cDNA, 2 μL, was used in a 10-μL qPCR volume with Fast SYBR Green Master Mix (Applied Biosystems) and diluted Qiagen validated human primers including *PDPN* (QT01015084), *CD248* (QT00216356), *CD106* (QT00018347) *β-actin* (QT00095431) and glyceraldehyde-3-phosphate dehydrogenase (*GAPDH*) (QT00079247). Duplicate reactions for each gene were run on a ViiA7 qPCR machine (Applied Biosystems) and results were calculated using the DDC_t_ method using reference genes for human β-actin and GAPDH. Results were consistent using these reference genes and data are shown normalized to β-actin.

### Flow cytometry

After harvest, tendon cells were washed twice in cell staining buffer (CSB) (BioLegend) and blocked for 15 minutes at room temperature (RT) in 20% human FcR blocking reagent (Miltenyi Biotech) diluted in CSB. All dead cells were excluded from analysis using fixable viability dye ef780 (1:1000 dilution) (eBioscience). Cells were stained in a buffer containing 20% FcR blocking reagent diluted in CSB at RT for 30 minutes. Antibody and isotype cocktails were prepared as indicated in Table 2. After washing, cells were fixed using Cytofix fixation buffer (BD Biosciences) for 20 minutes at RT. Flow cytometry was performed on a BD LSR Fortessa instrument calibrated daily with BD cytometer setup and tracking beads. Analysis of data was carried out using FlowJo software (Treestar).

**Table 2:** Antibodies used for flow cytometry

Antibody	Catalogue number	Clone
PDPN Alexa Fluor 488 anti-human	337005 BioLegend	NC08
Alexa Fluor 488 Rat IgG2aκ isotype	400525 BioLegend	RTK2758
CD248 Alexa Fluor 647 anti-human	564994 BD Biosciences	B1/35
Alexa Fluor 647 mouse IgG1κ isotype	557714 BD Biosciences	MOPC-21
CD106 PE anti-human	305806 BioLegend	STA
PE mouse IgG1κ isotype	400112 BioLegend	MOPC-21
CD34 PerCP/Cy5.5 anti-human	343521 BioLegend	581
PerCP/Cy5.5 mouse IgG1κ isotype	400149 BioLegend	MOPC-21
CD45 BV605 anti-human	304041 BioLegend	H130
BV605 mouse IgG1κ isotype	400161 BioLegend	MOPC-21

All antibodies were diluted 1:50 for staining

### Statistical analysis

Statistical analyses were performed using GraphPad Prism 6 (GraphPad Software). Normality was tested using the Shapiro-Wilk normality test. The Kruskal-Wallis test followed by pairwise the post hoc Mann-Whitney *U* test was used to compare *PDPN*, *CD248* and *CD106* mRNA expression in healthy, diseased and post-treatment tendons. The pairwise Mann-Whitney *U* test was used to test for differences in gene and protein expression of PDPN, CD106 and CD248 between vehicle and IL-1β-treated healthy and diseased tendon cells. The pairwise Mann-Whitney *U* test was used to test for differences between mRNA expression of *PDPN*, *CD106* and NF-κB target genes in cytokine-treated healthy and diseased tendon cells. *P* < 0.05 was considered statistically significant.

## Results

### Diseased tendon tissues express stromal fibroblast activation markers

Markers of stromal fibroblast activation including PDPN, CD248 and CD106 have not been investigated in healthy and diseased tendon tissues. Post-treatment supraspinatus tendon biopsy samples were collected from patients 2–4 years after surgical sub-acromial decompression (SAD) treatment. This post-treatment patient group consisted of six patients who were asymptomatic after treatment and five who remained symptomatic.

**Fig. 1:**
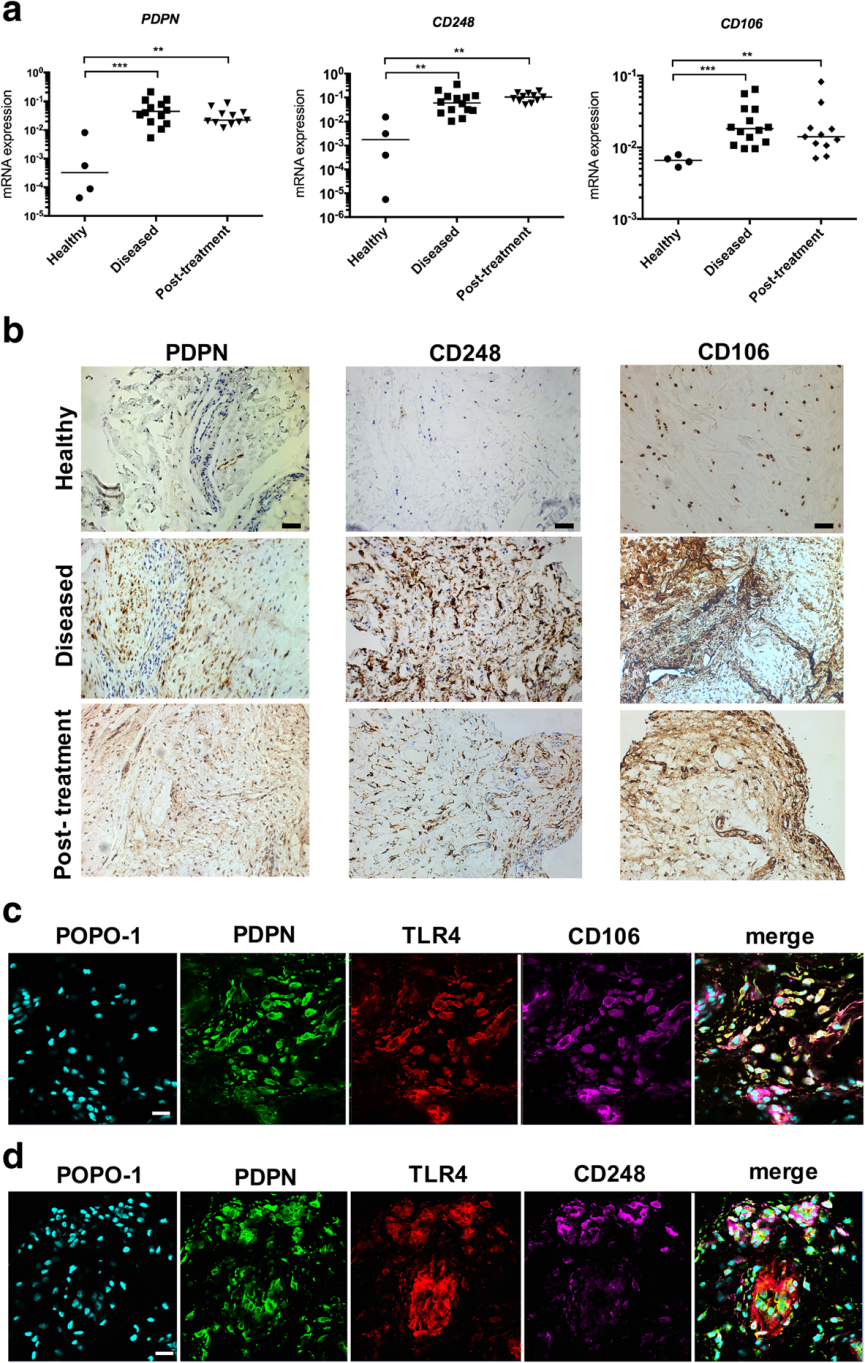
Diseased tendon tissues express markers of stromal fibroblast activation. **a** mRNA expression of stromal fibroblast activation markers *podoplanin* (*PDPN*), *CD248* and *CD106* in healthy subscapularis tendons and diseased and post-treatment supraspinatus tendons. *Bar* shows median values. Statistically significant differences were calculated using the Kruskal-Wallis test with the pairwise post hoc Mann-Whitney *U* test; **p < 0.01, ***p < 0.001. **b** Representative images of 3,3’-diaminobenzidine immunostaining (*brown*) for PDPN, CD248 and CD106 in healthy, diseased and post-treatment supraspinatus tendons. Nuclear counterstain is haematoxylin. *Scale bar* 50 μm. **c** and **d** Representative immunofluorescence images of sections of diseased supraspinatus tendons stained for markers of stromal activation (PDPN, *green*; CD248 and CD106, *purple*) and toll-like receptor 4 (*TLR4*) (*red*). *Cyan* represents POPO-1 nuclear counterstain. *Scale bar* 20 μm

Diseased supraspinatus tendons had significantly increased *PDPN, CD248* and *CD106* mRNA compared to healthy subscapularis tendons (Fig. 1a) (*p* = 0.001, *p* = 0.003 and *p* = 0.0007, respectively). This stromal activation signature was also present in supraspinatus tendon biopsies from post-treatment patients (*p* = 0.0015, *p* = 0.0015 and *p* = 0.006, respectively). Diseased and post-treatment supraspinatus tendons had increased immunopositive staining for PDPN, CD248 and CD106 compared to healthy supraspinatus tendons (Fig. 1b). Co-staining revealed co-localization of PDPN, CD106 and toll-like receptor 4 (TLR4) in diseased tendons (Fig. 1c). CD248+ cells were closely associated with clusters of PDPN+ cells. However, only a few cells expressed both PDPN and CD248 (Fig. 1d).

### Markers of stromal fibroblast activation are increased in diseased compared to healthy tendon-derived stromal cells

**Fig. 2:**
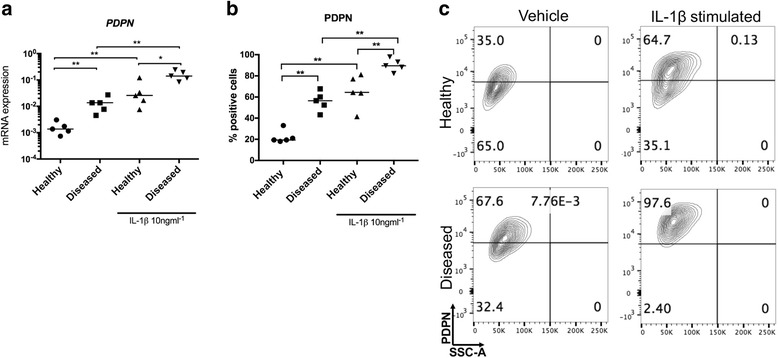
Expression of podoplanin (*PDPN*) mRNA and protein in healthy and diseased tendon cells after IL-1β treatment. Tendon cells were derived from healthy hamstring (n = 5 donors) and diseased supraspinatus tendons (n = 5 donors). Tendon cells were treated with medium containing vehicle only or IL-1β (10 ng/ml^-1^) for 24 hours. *PDPN* mRNA expression (**a**) and PDPN protein (**b**) are shown in healthy and diseased tendon cells after vehicle and IL-1β treatments. **c** Representative fluorescence-activated cell sorting (FACS) contour plots for PDPN from healthy and diseased tendon cells after vehicle and IL-1β treatments gated on CD45^-^CD34^-^ cells. *Bar* shows median values. Statistically significant differences were calculated using the Kruskal-Wallis test with the pairwise post hoc Mann-Whitney *U* test; *p < 0.05, **p < 0.01, ***p < 0.001

Markers of stromal fibroblast activation identified in diseased human tendons were further studied in healthy and diseased tendon-derived stromal cells to investigate the effects of cytokine treatment on stromal fibroblast activation in vitro. Diseased cells had increased *PDPN* mRNA compared to healthy cells under baseline unstimulated conditions (medium containing vehicle only) (*p* = 0.008) (Fig. 2a). IL-1β treatment of healthy and diseased cells for 24 hours further induced *PDPN* (*p* = 0.008). Induction of *PDPN* was more profound in IL-1β-treated diseased compared to IL-1β-treated healthy cells (*p* = 0.03). PDPN protein was increased in diseased compared to healthy cells under baseline unstimulated conditions (*p* = 0.008) (Fig. 2b and c). IL-1β treatment of healthy and diseased cells further induced PDPN (*p* = 0.008). Induction of PDPN was more profound in IL-1β-treated diseased compared to IL-1β-treated healthy cells (*p* = 0.008).

**Fig. 3:**
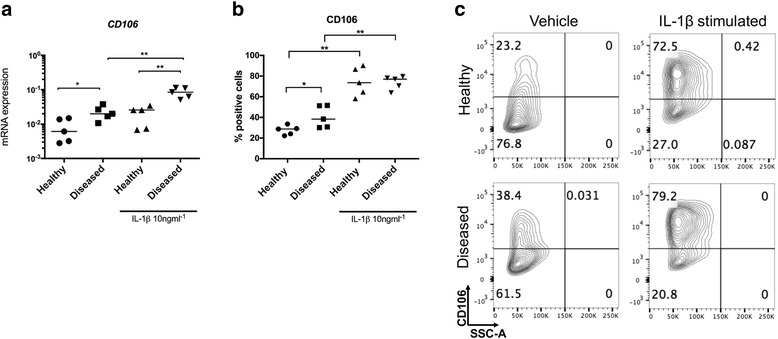
Expression of CD106 mRNA and protein in healthy and diseased tendon cells after IL-1β treatment. Tendon cells were derived from healthy hamstring (n = 5 donors) and diseased supraspinatus tendons (n = 5 donors). Tendon cells were treated with medium containing vehicle only or IL-1β (10 ng/ml^-1^) for 24 hours. *CD106* mRNA expression (**a**) and CD106 protein (**b**) are shown in healthy and diseased tendon cells after vehicle and IL-1β treatments. **c** Representative fluorescence-activated cell sorting (FACS) contour plots for CD106 from healthy and diseased tendon cells after vehicle and IL-1β treatments gated on CD45^-^CD34^-^ cells. *Bar* shows median values. Statistically significant differences were calculated using the Kruskal-Wallis test with the pairwise post hoc Mann-Whitney *U* test; *p < 0.05, **p < 0.01

Diseased cells had increased *CD106* mRNA compared to healthy cells under baseline unstimulated conditions (*p* = 0.03) (Fig. 3a). IL-1β treatment of diseased cells further induced *CD106* (*p* = 0.008). Induction of *CD106* was more profound in IL-1β-treated diseased compared to IL-1β-treated healthy cells (p = 0.008). CD106 protein was increased in diseased compared to healthy cells under baseline unstimulated conditions (*p* = 0.03) (Fig. 3b and c). IL-1β treatment of healthy and diseased cells further induced CD106 (*p* = 0.008).

**Fig. 4:**
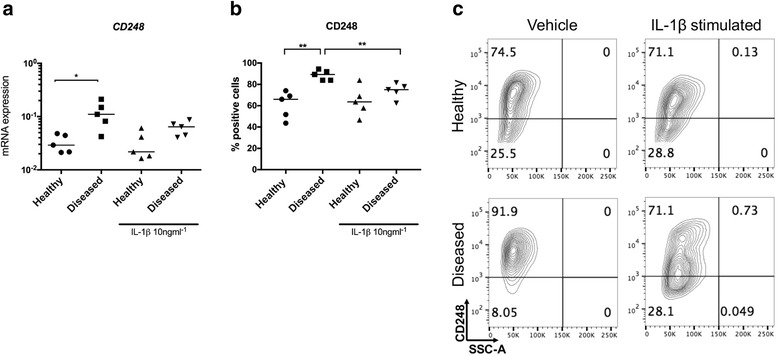
Expression of CD248 mRNA and protein in healthy and diseased tendon cells after IL-1β treatment. Tendon cells were derived from healthy hamstring (n = 5 donors) and diseased supraspinatus tendons (n = 5 donors). Tendon cells were treated with medium containing vehicle only or IL-1β (10 ng/mL^-1^) for 24 hours. *CD248* mRNA (**a**) and CD248 protein (**b**) are shown in healthy and diseased tendon cells after vehicle and IL-1β treatments. **c** Representative fluorescence-activated cell sorting (FACS) contour plots for CD106 from healthy and diseased tendon cells after vehicle and IL-1β treatments gated on CD45^-^CD34^-^ cells. *Bar* shows median values. Statistically significant differences were calculated using the Kruskal-Wallis test with the pairwise post hoc Mann-Whitney *U* test; *p < 0.05, **p < 0.01, ***p < 0.001

Diseased cells also had increased *CD248* mRNA compared to healthy cells under baseline unstimulated conditions (*p* = 0.03) (Fig. 4a). IL-1β treatment did not significantly attenuate *CD248* mRNA. CD248 protein was increased in diseased compared to healthy cells under baseline unstimulated conditions (*p* = 0.008) (Fig. 4b and c). IL-1β treatment of diseased cells reduced CD248 (*p* = 0.008).

### Inflammation induces persistent stromal activation in tendon-derived stromal cells

**Fig. 5:**
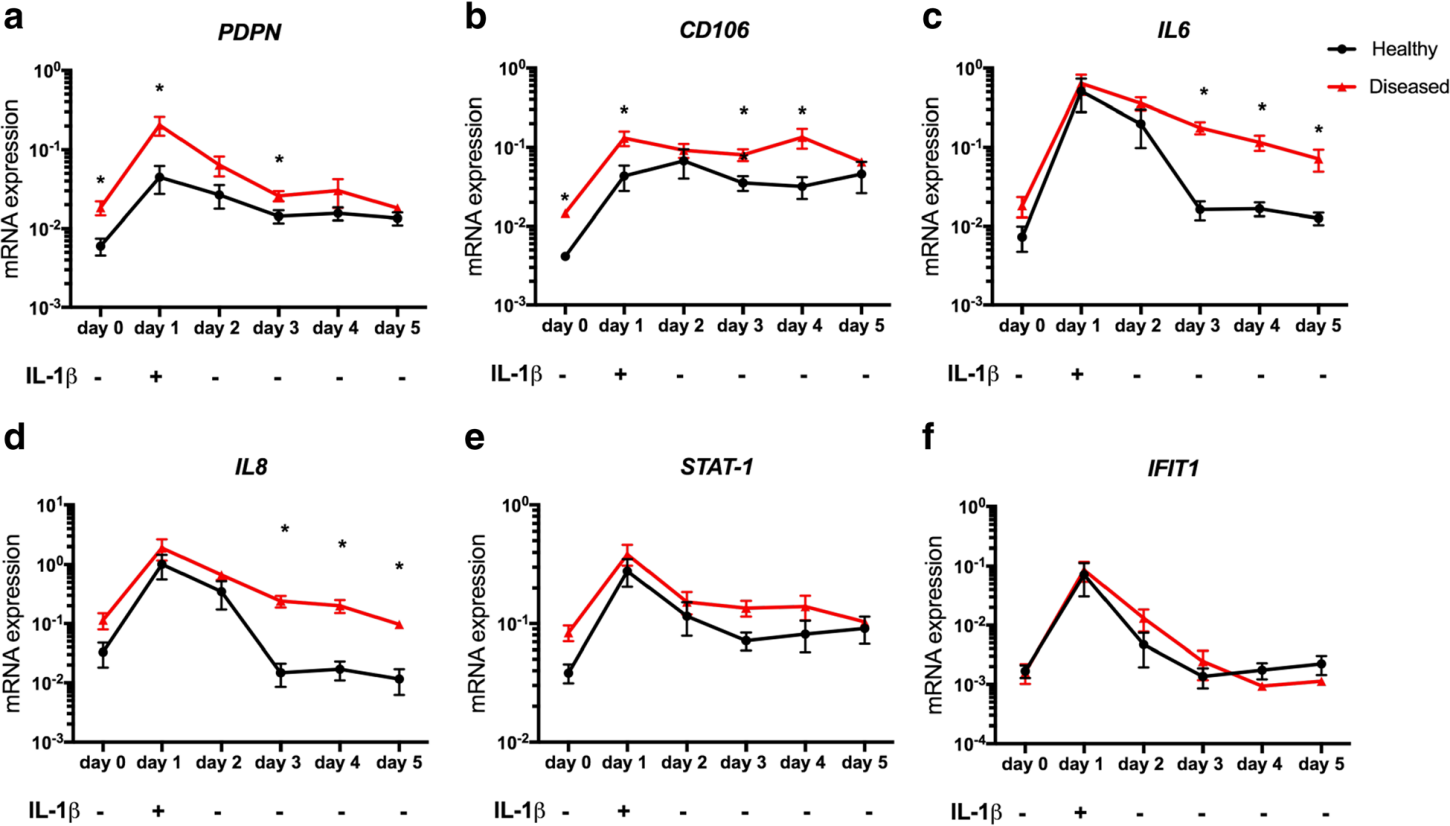
Effects of IL-1β on podoplanin (*PDPN*), *CD106* and NF-κB genes in healthy and diseased tendon cells over time. Tendon cells were derived from healthy hamstring (n = 4 donors) and diseased supraspinatus tendons (n = 4 donors). Cell lysates were sequentially harvested at day 0 (pre-stimulation), day 1 (24 hours after IL-1β treatment, 10 ng/mL^-1^) and days 2, 3, 4 and 5 when cells were incubated in cytokine-free medium containing vehicle only. mRNA expression is shown for *PDPN* (**a**), *CD106* (**b**), *IL6* (**c**), *IL8* (**d**), *STAT-1* (**e**) and *IFIT1* (**f**). Data are mean ± SEM. Statistically significant differences were calculated using the pairwise Mann-Whitney *U* test for healthy and diseased cells at each time point; *p < 0.05

Having identified that stromal fibroblast activation markers were present in diseased tendon tissues 2–4 years after treatment, we sought to investigate if IL-1β treatment induces stromal fibroblast “memory” in healthy and diseased tendon cells in vitro. Diseased cells had increased *PDPN* and *CD106* mRNA compared to healthy cells under baseline unstimulated conditions (*p* = 0.03 respectively). IL-1β treatment for 24 hours induced *PDPN* and *CD106*, induction was more profound in diseased compared to healthy cells (*p* = 0.03) (Fig. 5 a and b). In healthy cells *PDPN* and *CD106* mRNA remained elevated beyond pre-stimulated levels following replacement with cytokine-free medium for 4 days (*p* = 0.03). In diseased cells, PDPN returned to pre-stimulated levels; however, CD106 remained elevated beyond pre-stimulated levels following replacement with cytokine-free medium for 4 days (*p* = 0.03).

IL-1β treatment of healthy and diseased tendon cells also induced NF-κB target genes including *IL6, IL8, STAT-1* and *IFIT1* (Fig. 5c-f)*.* In healthy cells there was a gradual decline in these pro-inflammatory genes following replacement with cytokine-free medium. IL-1β-treated diseased cells had a more sustained induction of *IL6* and *IL8* compared to IL-1β-treated healthy cells between 2 and 4 days following replacement with cytokine-free medium (*p* = 0.03 for each respective time point) (Fig. 5c and d).

#### Positive and isotype controls for immunostaining

**Fig. 6:**
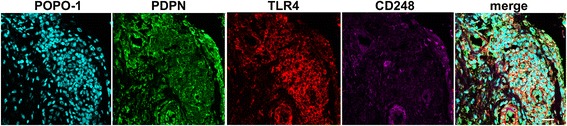
Positive control staining of rheumatoid synovium for markers of stromal fibroblast activation. Representative confocal immunofluorescence images show podoplanin (*PDPN*) (*green*), CD248 (*purple*) and toll-like receptor 4 (*TLR4*) (*red*). Cyan represents POPO-1 nuclear counterstain. *Scale bar* 20 μm.

**Fig. 7:**
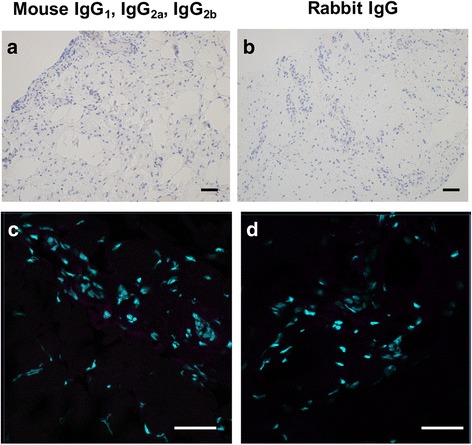
Isotype control staining of diseased human supraspinatus tendons. **a**, **b** Representative bright field images of diseased tendon sections stained with isotype control antibodies for mouse IgG_1_, IgG_2a_, IgG_2_b and rabbit IgG fractions. Nuclear counterstain is haematoxylin. *Scale bar* 50 μm. **c**, **d** Representative confocal immunofluorescence images showing merged image of diseased tendon sections stained with isotype control antibodies for mouse IgG_1_, IgG_2a_, IgG_2b_ and rabbit IgG fractions. *Cyan* represents POPO-1 nuclear counterstain. *Scale bar* 20 μm

Sections of diseased rheumatoid synovium were used as positive controls to confirm immunostaining for PDPN and CD248 (Fig. 6). Isotype control staining was performed using sections of diseased supraspinatus tendons (Fig. 7a and b).

## Discussion

The effect of the inflammatory process on resident tendon stromal cells is poorly understood. This study provides new insights into the pathogenesis of diseases affecting these musculoskeletal soft tissues. We identified a mechanism whereby diseased tendon tissues and cells become activated after exposure to an inflammatory stimulus, which induces a sustained change in their phenotype whereby cells express markers of stromal fibroblast activation including PDPN, CD106 and CD248. This longitudinal cohort study of phenotyped human tendon tissues collected pre and post treatment revealed that this stromal fibroblast activation signature persisted in tendon biopsies from patients who were up to 4 years post-treatment. Stromal activation and its persistence in vivo were further confirmed by IL-1β treatment of cultured tendon stromal cells in vitro.

Stromal fibroblast activation is a feature of the synovium in RA, where markers including PDPN, CD106 and CD248 have been identified [11, 12]. To our knowledge this is the first study to identify these stromal fibroblast activation markers in non-immune mediated musculoskeletal disease. IL-1β treatment of cultured tendon stromal cells induced markers of fibroblast activation including PDPN and CD106 but not CD248, suggesting distinct stromal responses in diseased tendons. In support of this, immunostaining of diseased tendons revealed co-localization of PDPN, CD106 and TLR4, suggestive of a pro-inflammatory tendon cell phenotype. CD248+ cells were closely associated with clusters of PDPN+ cells, but only a small number of cells expressed both PDPN and CD248 in diseased tendons. In RA, PDPN and CD106 are located in the synovial lining, and CD248 is located in the sub-lining layer, and these are thought to represent distinct fibroblast subsets [12, 13]. PDPN-expressing fibroblasts possess a pro-inflammatory phenotype in RA and malignancy [21, 22]. CD248-expressing fibroblasts have also been identified in hepatic and renal fibrosis, representing a reparative population [23].

Cancer-associated fibroblasts are known to maintain a state of permanent activation after the effects of the initiating stimulus subsides. This activated phenotype persists until cell senescence [24]. Cells populating diseased tendons are known to be apoptotic and senescent [5]. Furthermore, turnover of tendon collagenous matrix components is known to be low [25, 26]. This may account for the sustained expression of stromal fibroblast activation markers in tendon tissues from patients up to 4 years after treatment in both symptomatic and asymptomatic patients.

In our previous work investigating inflammation in cultured stromal cells derived from diseased human tendons, we proposed that diseased cells may be primed for inflammation [8]. In the current study, we identified IL-1β-treated diseased tendon cells showed a more profound induction of *PDPN* and *CD106* and sustained expression of *IL6* and *IL8 mRNA* compared to IL-1β-treated healthy tendon cells. Our findings support the concept that diseased tendon cells previously exposed to inflammation are primed and become hyper-responsive on subsequent exposure, possessing “stromal memory”. In support of this, studies of cancer-associated fibroblasts [27, 28] and of synovial fibroblasts in RA [9, 10] emphasize the important contribution of resident stromal cell populations to the persistence of chronic inflammation. Epigenetic changes in stromal cell populations are thought to be implicated in fibroblast activation. Changes in the epigenome can modulate the inflammatory response and promote the development of chronic disease [29].

Diseased tendon tissues investigated in the current study were collected from patients undergoing sub-acromial decompression surgery or surgical debridement of a supraspinatus tendon tear ranging from small (<1 cm) to massive (>5 cm) in size. Patients with large to massive tendon tears likely represent the end stage of the disease spectrum. Stromal fibroblast activation markers were highly expressed in all tendinopathic and torn tissues investigated in this study. We recently identified the plasticity and complexity of inflammation activation signatures in diseased human shoulder tendons [8]. The current study using tissues from these same patient cohorts demonstrated that markers of stromal fibroblast activation are highly expressed in diseased tendons, and that disease stage does not influence the degree of expression of PDPN, CD106 and CD248. These findings further support the concept of stromal fibroblast “memory” and suggest that exposure of tendon cells to an inflammatory stimulus induces a sustained change in their phenotype.

Repetitive damage through cumulative loading is a known contributor to the development of tendon disease. Mechanical cues can also induce tendon cells and tissues to release pro-inflammatory mediators including prostaglandins and pro-inflammatory cytokines [30, 31]. The effects of mechanical loading on stromal activation in tendinopathy have not been directly investigated. However, it is conceivable that in a disease setting, both mechanical and chemical cues may potentially induce stromal fibroblast activation as both stimuli possess the capacity to induce inflammation.

We acknowledge there are potential limitations with the use of hamstring tendons as a comparator to diseased tendons, including tendon type and age differences. However, hamstring tendon was taken from live healthy donors without history of tendinopathy. We believe this is a more suitable comparator than cadaveric shoulder tendon tissues, where little is known about whether the tendons were healthy or diseased or whether tendons were affected by post mortem changes.

## Conclusions

The findings from this study support the important and sustained contribution of diseased tendon stromal cells to the development and persistence of non-resolving tendon inflammation. We showed for the first time that stromal fibroblast activation markers are increased in diseased compared to healthy human tendon tissues and cells and identified distinct stromal responses in diseased tendons. We demonstrated that inflammation induces stromal fibroblast activation and stromal “memory”, which is more profound in diseased compared to healthy tendon cells. We propose that persistent stromal fibroblast activation is an important mechanism for the development of chronic inflammation and recurrent tendinopathy. Targeting activated tendon stromal cells is a potential therapeutic strategy for curative intervention.

## Abbreviations

BMI: body mass index
BSA: bovine serum albumin
CSB: cell staining buffer
DAB: 3,3’-diaminobenzidine
FCS: fetal calf serum
GAPDH: glyceraldehyde-3-phosphate dehydrogenase
GCR: glucocorticoid receptor
IFIT1: interferon-induced protein with tetratricopeptide repeats 1
IFN: interferon
IHC: immunohistochemistry
IL: interleukin
NF-κB: nuclear factor kappa beta
OSS: Oxford shoulder score
PBS: phosphate-buffered saline
PBST: phosphate-buffered saline with Tween
PDPN: podoplanin
RA: rheumatoid arthritis
RT: room temperature
RT-qPCR: real-time quantitative polymerase chain reaction
SAD: sub-acromial decompression
STAT: signal transducer and activator of transcription
TLR4: toll-like receptor 4
